# Starvation reveals the cause of infection-induced castration and gigantism

**DOI:** 10.1098/rspb.2014.1087

**Published:** 2014-10-07

**Authors:** Clayton E. Cressler, William A. Nelson, Troy Day, Edward McCauley

**Affiliations:** 1 Department of Biology, Queen's University, Kingston, Ontario, Canada; 2 Department of Mathematics and Statistics, Queen's University, Kingston, Ontario, Canada; 3 Department of Biological Sciences, University of Calgary, Calgary, Alberta, Canada

**Keywords:** host–parasite interaction, life history, castration, gigantism, starvation

## Abstract

Parasites often induce life-history changes in their hosts. In many cases, these infection-induced life-history changes are driven by changes in the pattern of energy allocation and utilization within the host. Because these processes will affect both host and parasite fitness, it can be challenging to determine who benefits from them. Determining the causes and consequences of infection-induced life-history changes requires the ability to experimentally manipulate life history and a framework for connecting life history to host and parasite fitness. Here, we combine a novel starvation manipulation with energy budget models to provide new insights into castration and gigantism in the *Daphnia magna*–*Pasteuria ramosa* host–parasite system. Our results show that starvation primarily affects investment in reproduction, and increasing starvation stress reduces gigantism and parasite fitness without affecting castration. These results are consistent with an energetic structure where the parasite uses growth energy as a resource. This finding gives us new understanding of the role of castration and gigantism in this system, and how life-history variation will affect infection outcome and epidemiological dynamics. The approach of combining targeted life-history manipulations with energy budget models can be adapted to understand life-history changes in other disease systems.

## 1. Introduction

Parasites often induce life-history changes in their hosts [1–3]. Some of these changes reflect changes in host behaviour [4], some are owing to direct physical damage caused by the parasite (e.g. toxin production), and some are related to host and parasite energetics [5]. The latter is because parasites, by definition, rely on host resources to fuel their replication. This dependence sets up an intimate and often antagonistic relationship between the parasite's resource requirements and the host's normal pattern of resource acquisition and allocation. Resource antagonism can have a direct impact on the life history of infected hosts [2]. It can arise from simple parasitic exploitation of resources reducing the amount of energy available for host physiological processes [6], from changes that the host makes to energy allocation in order to defend itself against parasitism [7], or from the parasite altering energy allocation to increase its access to resources [8]. As a result, changes in host life history can benefit the host, the parasite or neither, depending on the environment and species involved [9].

Understanding the proximate causes and consequences of infection-induced changes in host life history requires understanding first how hosts acquire and normally allocate resources, and second where parasites gain their energy along this allocation pathway. From this knowledge, we can connect observed life-history changes to fitness consequences for both the host and parasite, thereby gaining insights into the evolution of such changes as host or parasite strategies [5,6,10,11]. We have well-developed and empirically verified bodies of theory that provide a basis for understanding the energetics of uninfected hosts [12,13]. For most disease systems, however, we lack a basic understanding of the mechanisms parasites use to exploit within-host resources.

A well-studied example is the *Daphnia magna–Pasteuria ramosa* host–parasite system. *Pasteuria* has a parasitoid life-history strategy, transmitting between hosts only upon host death. Like many parasitoids, *Pasteuria* castrates its host before killing it [14]: several days after exposure, host reproduction stops (castration) and growth accelerates (gigantism). Once the host is castrated, assuming castration is permanent, the parasite has won the battle over within-host resources [15]. However, prior to castration, infected hosts often have larger clutches than same-age uninfected individuals, a host adaptation known as fecundity compensation [8,16]. Moreover, the timing of castration (i.e. the length of the lag between exposure and the final host reproductive bout) has important implications for both the host and the parasite: the longer it takes for the parasite to castrate its host, the higher is host reproduction and the lower is parasite spore production [8,17]. These observations suggest that energy antagonism has significant effects on both host and parasite fitness, even in a castrating system.

Energy budget models provide a framework for hypothesizing how castration and gigantism arise out of the interaction between host energetics and parasite exploitation [11]. These models describe the dynamics of growth and reproduction as outcomes of the processes of resource acquisition and within-host allocation [12]. By modifying these models to include epidemiological processes that require energy, such as parasitic exploitation or immune activation, we can use them to study infection-induced life-history changes [6]. This allows us to make predictions about how variation in host energy budget parameters, such as ingestion rate, relative allocation to growth versus reproduction or somatic maintenance rate, will contribute to variation in disease processes such as infection success, parasite within-host growth rate and host immune response [18,19]. We can also predict how variation in the external resource environment will affect these processes [20].

**Figure 1.**
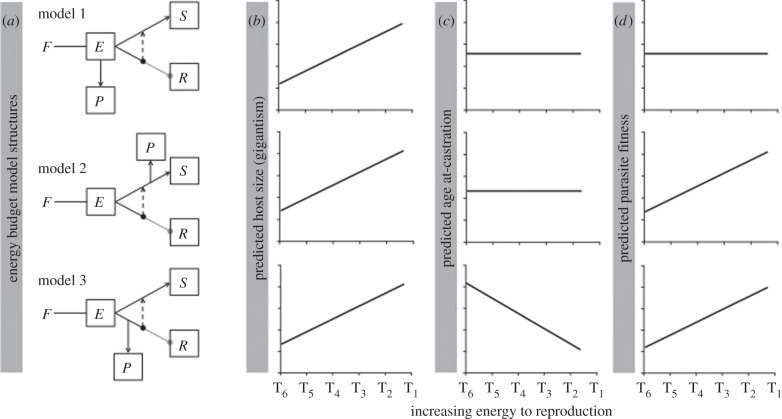
Predicted host and parasite response to changing energy flow to host reproduction. (*a*) Alternative energy budget models for parasite growth in the *D. magna*–*P. ramosa* system. These models are based on the dynamic energy budget framework of [16]. *F* is food in the environment; *E* is energy reserves, a temporary storage buffer for assimilated energy; *S* is somatic tissue; *R* is reproductive investment; and *P* is the parasite population. In all models, infection causes energy to be reallocated from reproduction to growth, as indicated by the dashed arrows. *Model 1* posits that parasites use the reserves as a resource [15], *model 2* posits that parasites use growth allocation as a resource [11] and *model 3* posits the parasites use reproduction allocation as a resource, with any surplus reallocated to growth. (*b–d*) Responses of host life history and parasite fitness to an experimental treatment that changes the energy flowing to reproduction without affecting the energy to growth. (*b*) In all models, increasing the energy to reproduction increases host size. (*c*) Models 1 and 2 predict no response of age at castration to the treatment, whereas model 3 predicts a more rapid onset of castration. (*d*) Model 1 predicts no response of parasite fitness to treatment, whereas models 2 and 3 predict that parasite fitness will increase. The label T_d_ denotes the feeding interval in days (d) used to manipulate host reproduction.

Previous authors have proposed proximate energetic explanations for how castration and gigantism arise in infected individuals [8,11], but there are several ways this could occur, and model predictions have never been directly compared against one another or against empirical data. Figure 1*a* shows three heuristic energy budget model structures for this system. These models are based on the framework of dynamic energy budget theory [12] and share broad similarities. In particular, under all models, castration and gigantism benefit the parasite [8,21]. Following castration, energy not captured by the parasite goes into host growth. The timing of castration is hypothesized to depend on the parasite's population growth rate (prior to castration). This is because castration is likely to be hormonal [8,14]; if hormone production is density-dependent, then castration occurs when the parasite population reaches some critical size [11]. The critical distinction among the models is in their assumption about where, in the normal host energy budget, the parasite gets its energy. Thus, comparing model-predicted responses to an experimental perturbation of energetics against empirical data can reveal which model structure is most appropriate for this system. In particular, consider an experimental treatment that holds total resource ingestion constant but reduces the amount of energy going to reproduction without affecting the amount of energy going to growth. The models predict very different responses of host gigantism, timing of castration and lifetime parasite fitness to such a treatment (figure 1*b–d*).

*Model 1*, proposed by Hall *et al.* [11], hypothesizes that the parasite uses energy stored in ‘reserves’ [12] as a resource. These reserves serve as a temporary storage buffer between food in the environment and host metabolic processes, such as growth and reproduction. Because the parasite has access to all of the ingested resources, and the treatment does not affect total ingestion, the parasite's population growth rate will be unaffected by treatment. Thus, the model predicts that age at castration (figure 1*c*) and parasite fitness (figure 1*d*) will be constant across a gradient of energy to reproduction. Gigantism, on the other hand, should increase with increasing energy to reproduction as, post-castration, there will be more energy available for growth (figure 1*b*).

*Model 2*, proposed by Ebert *et al.* [8], hypothesizes that the parasite uses energy going to growth as a resource. The energy liberated by castration becomes available both for parasite population growth and for host somatic growth. The model predicts that increasing the amount of energy to reproduction will increase both host growth and parasite fitness (figure 1*b,d*). However, because the amount of energy going towards growth prior to castration is the same across treatments (electronic supplementary material, figure S4), the model predicts age at castration will be constant (figure 1*c*).

*Model 3* has not previously been considered, but nicely mirrors model 2, providing a useful comparison. In this model, the parasite uses energy going to reproduction as a resource. Castration occurs through a combination of parasite exploitation and hormonally controlled energy reallocation. Any surplus energy that makes it past the parasite is reallocated towards growth. As with model 2, this model predicts that increasing the amount of energy going to reproduction will increase host growth and parasite fitness (figure 1*b,d*). However, the age at castration will decrease as the pre-castration parasite population growth rate will increase with increasing energy to reproduction (figure 1*c*).

Here, we use a novel starvation experimental design to reveal the structure of the internal energetic interaction in the *D. magna–P. ramosa* host–parasite system. In *Daphnia*, previous work has found that increasing the duration of starvation results in decreased reproduction, but with little impact on growth [22]. We leverage this aspect of *Daphnia* biology to manipulate the energy to reproduction, and test where *Pasteuria* gains its energy. The treatments provide the same total amount of food, but vary the time interval between feedings. Increasing the interval between feedings was found to reduce the amount of energy going to reproduction in uninfected animals without affecting growth. Thus, comparing observed host growth and reproduction and *Pasteuria* spore production with the predictions of the heuristic models (figure 1) will allow us to determine which model is best supported.

## 2. Methods

*Daphnia magna* is a cyclical parthenogenetic crustacean found in freshwater ponds. The clone (KA30) used in this study was collected from a pond at Kaimes Farm, Leitholm, Scottish Borders. *Pasteuria ramosa* is a bacterial endoparasite found to infect several species of *Daphnia*, including *D. magna*. Transmission occurs via feeding, with *D. magna* consuming bacterial spores that have been released into the water column from dead hosts. The isolate of *P. ramosa* used in this study came from a single infected *D. magna* from the Kaimes Farm population.

Prior to the experiment, individual *D. magna* were raised in 35 ml glass vials containing 20 ml of phosphorus- and nitrogen-replete COMBO medium [23]. Removing these macronutrients helps prevent bacterial contamination without affecting host life history [24]. Individuals were maintained at 20°C and fed 0.1 mg C day^−1^ of the green alga *Chlamydomonas reinhardtii*. Algae were cultured in 1 l flasks containing 500 ml of high nitrogen COMBO medium at 23°C under a 16 : 8 light–dark cycle. The high-nitrogen COMBO medium was created by doubling the nitrogen in the base medium. On the sixth day post-inoculation, algae was concentrated using a centrifuge, and cell density was counted in a Sedgwick cell under a compound microscope. We used a cell carbon content of 40 pg C based on measured cell volume [25].

After feeding, *Daphnia* were kept in the dark to prevent algal growth, which allows for strict control of total ingestion [24]. The clone line was maintained under these conditions for three generations to standardize maternal effects. From these standardized individuals, 360 *D. magna* neonates were introduced into the experiment within 24 h of birth. These individuals were randomly placed into one of six feeding treatments (described below), with 40 individuals within each feeding treatment randomly assigned to be exposed to the parasite and 20 individuals maintained as controls.

As with the maternal lines, individuals were maintained in 35 ml glass vials, fed 0.1 mg C day^−1^ of algae and transferred every 3 days to fresh medium until day 9 of the experiment. At each transfer, the length of each animal was measured under a dissecting scope, and the neonates produced during the intertransfer interval were counted. Length was measured from the base of the tail spine to the top of the head. *D. magna* does not grow continuously, but rather changes its length when it moults its outer carapace. The moult period of *D. magna* is approximately 3 days and we never observed more than a single moulted carapace in the vial, so our measurement interval was sufficient to capture each moulting event.

On day 9 of the experiment, all of the individuals assigned to the exposed class (240 in total) were exposed to 250 000 spores of the *P. ramosa* isolate. These spores were collected from 70 previously infected individuals of the same genotype, who were raised under conditions identical to the maternal lines in this experiment. To harvest spores from these animals, each individual was homogenized in 500 μl of double-distilled water using a Kontes Pellet Pestle cordless motor and pestle. The homogenates from all 70 individuals were combined, and the spore density was determined by counting transmission-stage spores using a haemocytometer and a compound microscope under 400× magnification. An appropriate amount of homogenate was then added to each vial to ensure that each individual was exposed to 250 000 transmission-stage spores. All of the individuals assigned to the control class (120 in total) were exposed to an identical volume of homogenate created by processing 70 unexposed *D. magna* from the same genotype in the same way. From day 9 to day 12, individuals were fed every day, as before, and stayed in the vial with spores. On day 12, individuals were removed from their vials, rinsed in medium to remove any spores attached to the carapace, and transferred to a clean vial containing the proper amount of food for their particular treatment. This ensures that differences in infection success cannot be attributed to differences in exposure, but only to the post-exposure effect of feeding treatment.

The six treatments varied the feeding interval from 1 to 6 days and correspondingly varied the transfer concentration of the food from 0.1 to 0.6 mg C per vial to ensure that total food available was constant across the treatments (electronic supplementary material, table S1). These food amounts were chosen based on a prior determination that 0.6 mg C of algae could be eaten (more than 97%) in a single day by a healthy 12-day-old *D. magna*. Thus, increasing the interval between feedings will increase the amount of starvation stress, because the animals will consume all of the food in a single day and then be without food until the next feeding. Individuals were fed according to this schedule, but were still measured and transferred every 3 days. This led to occasions when the animals were fed and then transferred 24 h later. To ensure that animals had equal access to food, prior to each transfer we estimated the concentration of algae remaining in each treatment by taking 100 μl samples of the medium from 10 randomly chosen vials within each treatment. The concentration of algae in this sample was used as an estimate of the amount ingested by the animals. If this concentration was less than 5% of the initial (feeding) concentration, then all animals in the treatment were transferred to vials containing new medium; otherwise, the medium was saved, and animals were measured and replaced in the same medium. We found animals in treatments T_1_–T_3_ always ate all of the algae prior to transfer, but animals in T_4_–T_6_ occasionally required more time, especially as the experiment progressed.

Individuals were maintained in their respective feeding treatments until natural death or day 42, at which point we terminated the experiment. All exposed individuals were placed in 1 ml microcentrifuge tubes and homogenized in 100 μl of double-distilled water. The homogenate was further diluted to a final volume of 500 μl. *Pasteuria ramosa* transmission-stage spores were counted as above. Each animal was homogenized individually using a unique pestle, and the haemocytometer was rinsed in ethanol between each count to guard against cross-contamination. Replicate counts of transmission spores were performed for each animal.

### (a) Data management

For all of the statistical analyses and results reported in the paper, we use only the data from individuals that lived at least 27 days. This was done because day 27 was the earliest we were able to detect infection in any of the exposed *Daphnia*, and therefore determine their infection class. We therefore use the growth and reproduction trajectories of control animals that lived at least 27 days to correspond with the data treatment of the exposed animals. This does affect our sample sizes, as increasing the interval between feedings also increased mortality (electronic supplementary material, figure S7). Growth and reproduction data were converted to the scale of carbon content to facilitate direct comparison. Length (L) was converted using the length–dry weight (DW) regression: log(μg DW) = 2.467 + mm L^2.767^, with 0.48 mg C per mg DW [26,27]. It is known that food environment has no effect on this regression [28]. Moreover, it should give a measure of the weight of host tissue only, as the stiff carapace prevents host length from changing due simply to the host ‘swelling up’ with spores. Eggs were converted to carbon content using a dry weight of 8 × 10^−3^ mg egg^−1^, with 0.57 mg C mg DW^−1^ [27]. Transmission-stage spores were converted to carbon content assuming a dry weight of 33.0 × 10^−9^ mg spore^−1^, with 0.48 mg C mg DW^−1^ [27].

### (b) Statistical analyses

Owing to gigantism and castration, the growth and reproduction trajectories cannot be described using traditional parametric equations. Thus, to analyse whether feeding treatment or infection class (control, exposed but uninfected, and infected) affected these trajectories, we used generalized additive mixed models (GAMMs) [29] that model them using smooth regression splines. GAMMs allow the relationship between age and growth or reproduction to better match the observed trajectories and differences in trajectory shape across treatments and be evaluated statistically. The linear mixed-effects component accounts for variation among individual *Daphnia*, which are a random subsample of the total population, and the fact that each individual was measured repeatedly. GAMM analysis was performed using the mgcv package in the R statistical software program [30]. The GAMM results are shown in the main figures, and all raw growth and reproduction trajectories are shown in the electronic supplementary material, figures S1 and S2. Infection success, the proportion of exposed animals living to day 27 that became infected, was evaluated using logistic regression; parasite fitness, measured by total spore production, host age and size across treatments, was tested using a log-linear model; and parasite fitness and host age at castration were evaluated using ANOVA. All statistical analyses were performed in the R statistical software program [30].

## 3. Results

**Figure 2.**
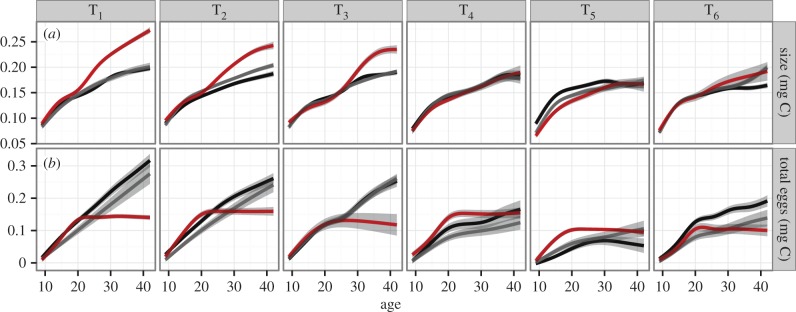
Host growth and reproduction across feeding interval treatment and infection class. Both axes are shown in cumulative carbon units to facilitate a direct comparison. Solid lines show the GAMM predictions for each treatment and class, and shadings show the standard error. (*a*) Feeding treatment has little effect on host growth in control (black) and uninfected animals (grey), but gigantism is substantially reduced in infected animals (red). (*b*) Feeding treatment substantially reduces host reproduction in control and uninfected animals, but has little impact on castration (the age when reproduction ceases) in the infected class.

Changing the feeding interval from 1 to 6 days had a minimal impact on the growth trajectories in the control animals (figure 2*a*; electronic supplementary material, table S2), but caused a strong decrease in the amount of reproduction (figure 2*b*; electronic supplementary material, table S2). For example, in the 4-day feeding interval treatment, the animals were 7% smaller on average, but reproduced 48% less on average (electronic supplementary material, figure S5). As anticipated, infection induced both gigantism and castration in the everyday feeding treatment (figure 2; electronic supplementary material, table S4). Infected animals also had larger clutches than uninfected animals, though this difference was only statistically significant in T_1_ and T_2_ (electronic supplementary material, figure S6). However, the magnitude of gigantism was reduced and its onset delayed as the feeding interval increased. The growth trajectories of control and infected animals were statistically indistinguishable in feeding treatments T_4_–T_6_ (figure 2*a*; electronic supplementary material, table S4). The exposed but uninfected animals had similar growth and reproduction trajectories to the control animals (electronic supplementary material, table S3).

**Figure 3.**
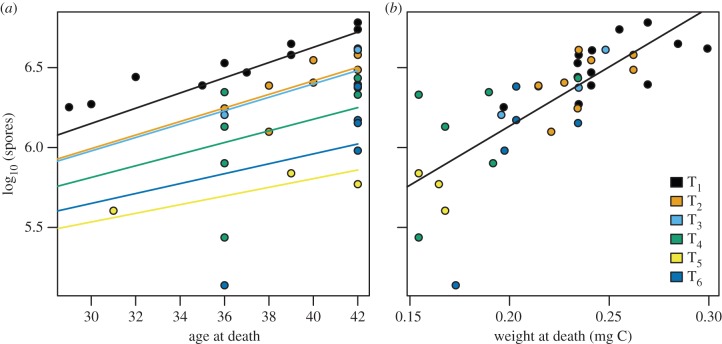
Parasite fitness across treatments for animals with transmission spores. (*a*) Parasite spore density increases with host age at death and decreases with reduced energy to host reproduction. (*b*) Parasite spore density increases with size of the animal, but this relationship is not affected by the manipulation of energy to host reproduction. Circles show parasite density of each animal at death, lines show the relationships with statistical support and colours denote the feeding interval treatment.

Infection success decreased slightly with increasing feeding interval, but this effect is not statistically significant (table 1; logistic regression, likelihood ratio test *χ*^2^ = 6.19, d.f. = 5, *p* = 0.29). Feeding treatment did have a significant effect on spore production. We expected spore load to increase with age at death [17]. To evaluate whether there was a significant effect of treatment on spore production, we fitted an exponential growth model to the spore load by age at death data. We found strong statistical support for feeding treatment affecting the rate of spore production (a significant effect of treatment on the slope of the log-linear regression; *F* = 10.93, d.f. = 5.32, *p* = 3.4 × 10^−6^; figure 3). We also expected spore load to increase with host size at death [8]. Interestingly, however, while spore load did increase with host size, this relationship was unaffected by feeding treatment (log-linear regression, *F* = 1.11, d.f. = 5.32, *p* = 0.37).

**Table 1.** Number of individuals surviving to day 28 in each infection class. Each treatment was started with 20 control and 40 exposed animals.

treatment	control	uninfected	infected	infection success (%)
T_1_	17	18	13	42
T_2_	14	21	9	30
T_3_	15	23	4	15
T_4_	12	16	6	27
T_5_	6	12	3	20
T_6_	8	12	6	33

**Figure 4.**
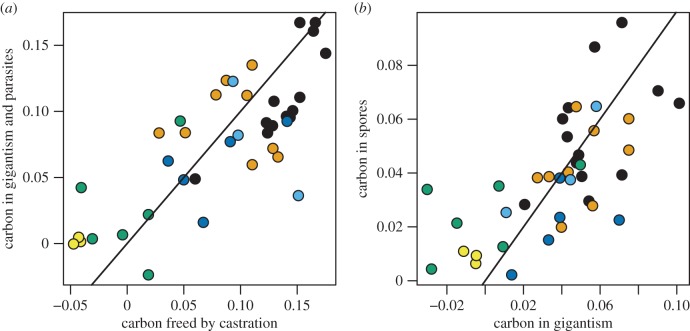
Carbon accounting in infected animals. (*a*) The solid line shows the 1 : 1 relationship between the expected carbon freed up by castration and the observed carbon in gigantism and parasite spores. (*b*) The solid line shows the 1 : 1 relationship between observed carbon in gigantism and in spores. Colours denote the feeding interval treatment as in figure 3.

Simple carbon accounting further illustrates the connection between host size and spore load. Figure 4 shows the relationship between the amount of carbon ‘liberated’ by castration and the amount of carbon observed in gigantism and spores. The carbon liberated by castration is calculated as the difference between the expected amount of carbon in eggs for a control animal in each treatment and the observed carbon in eggs for each infected animal. The amount of carbon in gigantism is calculated as the difference between the observed size of each infected animal and the expected size for a control animal in each treatment. Across feeding treatments, the data show a strong correlation between the amount of carbon liberated by castration and the amount ending up in extra host tissue and spores (figure 4*a*; Pearson's *r* = 0.77). The data also suggest that host growth and parasites end up with nearly equal amounts of liberated carbon (figure 4*b*; Pearson's *r* = 0.68). The black line shows the one-to-one relationship in both panels of figure 4.

**Figure 5.**
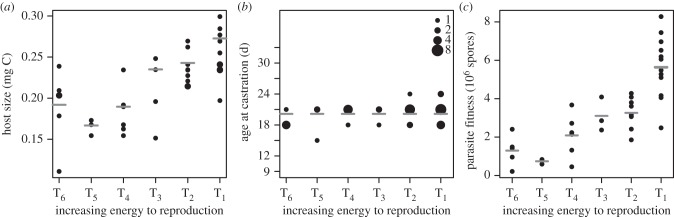
Observed host and parasite responses to changing energy to host reproduction. (*a*) Host size increases with increased energy to host reproduction. Points show the observed weight at death, with grey lines showing the expectation based on GAMM fits (figure 2*a*). (*b*) Age at castration shows no response to changes in energy to host reproduction. In (*a*,*b*), symbol area denotes number of individuals with observed weight or age at castration. (*c*) Parasite spore load increases with increased energy to host reproduction. Observations of parasite fitness were made at death of the animal. Because the treatments had an influence on survivorship, we corrected parasite fitness for differences in survivorship by scaling each individual to a common age of 42 days using the treatment-specific spore by age-at-death relationships shown in figure 3*a*. The label T_d_ denotes the feeding interval in days (d) used to manipulate host reproduction. Grey lines show the trends with statistical support across treatment.

We can relate our results back to the predictions made by the energy budget models. Host size increased with increasing energy to reproduction (figure 5*a*). Age at castration was unaffected by treatment (figure 5*b*; ANOVA, *F* = 1.14, d.f. = 5.32, *p* = 0.36), despite the fact that the treatments caused an observable change in pre-castration fecundity (electronic supplementary material, figure S3). Parasite fitness increased with increasing energy to reproduction (figure 5*c*; ANOVA, *F* = 18.0, d.f. = 5.32, *p* = 1.38 × 10^−8^).

## 4. Discussion

We set out to determine the structure of within-host energy utilization during infection in the *D. magna*–*P. ramosa* system. Our experimental design employed a novel starvation manipulation that reduced the energy flow to reproduction without affecting the energy flow to growth (figure 2; electronic supplementary material, figures S3 and S4), and then tracked the consequences of that manipulation for host gigantism and castration, and parasite spore production. This manipulation delayed and decreased the magnitude of gigantism, such that infected animals in treatments T_4_–T_6_ had growth dynamics that were statistically indistinguishable from those of control animals (figures 2*a* and 5*a*; electronic supplementary material, table S4). However, reducing the energy to reproduction had no effect on castration, with age at castration being the same across treatments (figure 5*b*). Parasite fitness was sharply reduced by decreasing energy to reproduction (figures 3 and 5*c*).

Comparing these results against the predictions of the three heuristic energy budget models laid out in the Introduction (figure 1), our experimental results clearly support model 2. Additional support for model 2 over model 3 comes from the observation that our experimental manipulation essentially induced reproductive castration in control animals in treatments T_5_ and T_6_ (figure 2*b*). This manifests itself in some of the infected animals reproducing more than the expectation based on the controls, leading to negative estimates of energy liberated by castration (figure 4). However, we still observe infection in these treatments, indicating that the parasite is getting energy from somewhere other than reproduction.

An energy budget perspective provides new insights into how variation in energy allocation will affect host and parasite fitness. Under model 2, a host that increases the allocation to reproduction and reduces the allocation to growth early in infection will have higher fitness. This is for two reasons: first, higher reproduction allocation will cause faster maturation and larger clutches prior to castration (fecundity compensation), as seen in our data and in previous experiments [8,16]; second, lower growth allocation will reduce the parasite's initial growth rate, delaying the onset of castration. Furthermore, there is some evidence that hosts can recover from infection [21]. If so, those most likely to recover will be the hosts with low parasite burdens, providing another benefit to reduced growth allocation. If fecundity compensation can indeed reduce the parasite's growth rate, then it functions as both a tolerance and a resistance mechanism.

This hypothesis is supported by the results of experiments that manipulated host energy allocation through exposure to fish kairomones [31]. *Daphnia* exposed to fish kairomones increase allocation to reproduction and reduce allocation to growth [32]. Therefore, hosts that have been exposed to kairomones prior to being exposed to the parasite are essentially pre-emptively carrying out the fecundity compensation allocation strategy. Model 2 would therefore predict that parasites infecting kairomone-exposed hosts should have lower initial growth rates than parasites infecting control hosts. This would delay the onset of castration and, consequently, host growth acceleration. Following castration, kairomone exposure should have no effect on parasite reproduction or host growth, as all of the energy is flowing to growth or the parasite. However, the initial reduction in parasite reproduction and the delay in gigantism imply that, at any fixed age, hosts exposed to kairomones before infection should be smaller and have lower parasite burdens. These predictions have been confirmed experimentally [31]. Moreover, Coors & de Meester [31] found that the negative effect of kairomones on spore production could be explained entirely by the reduction in growth, echoing our results (figures 3*b* and 4*b*).

We can also use the energy budget structure to understand how castration and gigantism benefit the parasite. Under model 2, the parasite uses castration to increase its access to resources. Because a constant fraction of the resources liberated by castration is used by the parasite (figure 4*b*), parasite fitness is determined by the efficiency of castration. However, gigantism may benefit the parasite in environments where resources are not limited, as ingestion rate is size-dependent. In such environments, larger hosts will have more energy flowing to growth per unit time. Compare this interpretation of the roles under either of the other models. In models 1 and 3, gigantism increases the parasite's access to resources by increasing ingestion, and thus the flow of energy into reserves (model 1) or reproduction (model 3). Castration in these models may be seen as an expedient mechanism for producing gigantism.

Our results suggest a way forward for conclusively demonstrating the adaptive value of gigantism. In particular, if *Pasteuria* strains differ in the fraction of liberated resources used by the parasite (figure 4*b*), then they differ in the relative allocation to (host) growth versus (parasite) reproduction, and standard life-history theory can be applied [33]. For a castrated host, selection is acting only on the parasite, as the host has no residual fitness [14], so we need only consider how additional host growth will affect the parasite's reproduction. Hechinger [15] has applied this theory to a system where a single host species is parasitized by several species of castrating parasites. Hechinger found that expected reproductive lifespan was longer for parasites than for uninfected hosts, and that, across parasite species, there was a strong positive correlation between expected reproductive lifespan and allocation to growth, exactly as predicted by standard life-history theory.

If *Pasteuria* strains do vary in their allocations to host growth versus parasite reproduction, then the adaptive value of parasite-induced growth changes would be demonstrated by showing that strains with low allocation to host growth have higher spore loads than strains with low allocation to growth early in infection, but that this pattern reverses later in infection. Some evidence for adaptive gigantism comes from selection experiments [34]. After five generations of selection for either fast development (early killing of hosts) or slow development (late killing of hosts), *Pasteuria* strains selected for fast development had higher spore loads early in infection than strains selected for slow development, but that pattern reversed later in infection. Assuming that development rate depends on the relative allocation to parasite reproduction, these results are in line with the predictions of life-history theory. If, however, *Pasteuria* strains do not vary in their allocations, then gigantism is merely a by-product of castration with no independent adaptive value.

Here, we used simple energy budget models inspired by dynamic energy budget theory [11,12] to characterize the energetics of a host–parasite system. Of course, more complex energetic models could be considered. For example, the parasite could potentially acquire energy from multiple locations, such as reserves and reproduction. Given our experimental treatments, if *Pasteuria* gets any energy from reproduction, the predictions will mirror those of model 3. If it gets energy from both reserves and growth, the predictions would be the same as for model 2. However, the strong correlation between liberated carbon and carbon in spores and host tissue suggests that the simpler model is likely to be sufficient. One could also consider additional pathways for energy, such as locomotion [13] or an immune response [20]. It is unclear whether such additions would provide alternative explanations for this system, as we do not know whether starvation or infection affect swimming behaviour and the role of the immune response is unclear in this system [35,36]. However, it is likely that such considerations will be important in other systems.

Parasites and hosts are generally in conflict over within-host resources. Given that host life history is the cumulative product of resource assimilation, allocation and usage over the organism's lifetime, it is unsurprising that parasitic infection influences host life-history traits [1–3]. However, it is often unclear who benefits from these changes [9]. Moreover, because both host and parasite fitness are affected by host life history, almost any possible host life-history response to infection (increased/decreased reproduction, increased/decreased size, increased/decreased mortality) can, in theory, benefit either the host or the parasite [5,37]. Here, we used a combination of novel experimental manipulations to perturb within-host energetics and heuristic energy budget models to understand the dynamics of castration and gigantism in the *D. magna*–*P. ramosa* system. This approach has given us new insights into how energetics influences both host and parasite fitness. In particular, the energetic structure suggests how fecundity compensation might act as both a tolerance and a resistance mechanism, and the ultimate evolutionary origin of castration and gigantism for the parasite. This approach could be adopted to understand the causes and consequences of host life-history change in other systems [6]. Doing so will increase not only our understanding of infection-induced host life-history changes, but also the ecological and evolutionary dynamics of disease more generally.

## Supplementary Material

Supplementary Tables and Figures
