# Supplementary material for: Starvation reveals the cause of infection-induced castration and gigantism
Source: Proc Biol Sci. 2014 Oct 7;281(1792):20141087. doi: 10.1098/rspb.2014.1087 (PMC4150321; doi:10.1098/rspb.2014.1087)
Supplement: Supplementary Tables and Figures [file rspb20141087supp1.docx]

**Supplementary Material**

**Figure S1:** Raw growth trajectories across feeding interval treatments. Axes are shown in cumulative carbon units to facilitate the comparison between growth and reproduction. The top row is control animals (black lines), the middle row is uninfected animals (gray lines) and bottom row is infected animals (red lines). The label $T_{d}$ denotes the feeding interval in days (d) used to manipulate host reproduction.

**Figure S2:** Raw reproduction trajectories across feeding interval treatments. Axes are shown in cumulative carbon units to facilitate the comparison between growth and reproduction. The top row is control animals (black lines), the middle row is uninfected animals (gray lines) and bottom row is infected animals (red lines). The label Td denotes the feeding interval in days (d) used to manipulate host reproduction.

**Figure S3:** Pre-castration reproduction (day 12 to day 24) across feeding treatments. We consider only clutches produced after the start of the treatments (Day 12). Because reproduction observed at a transfer represent prior energy investment, neonates observed on day 24 represent the clutch that is immediately after the mean age of castration. The response of pre-castration fecundity to the treatments was not different for control versus exposed uninfected animals (ANOVA, F=1.83, df=6,188, p=0.096). Both the control and uninfected animals show a significant decrease in pre-castration reproduction with an increase in the feeding interval (ANOVA, F=7.12, df=5,188, p=4.01e-6). Control animals are shown in dark grey and uninfected in light gray. Asterisks denote statistically significant differences in pre-castration reproduction from $T_{1}$ for both control and uninfected animals.

**Figure S4:** Pre-castration growth (day 12 to day 24) across feeding treatments. Pre-castration growth was not different across treatment (ANOVA, F=1.46, df=5,188, p=0.205) or between control and exposed but uninfected animals (ANOVA, F=1.59, df=1, 192, p=0.209). Control animals are shown in dark grey and uninfected animals in light gray.

**Figure S5:** Expected carbon investment in growth (black circles) and eggs (gray squares) by day 42 for a control animal in each of the six feeding treatments. Expectations are based on GAMMs fit to each feeding treatment separately.

**Figure S6:** Size of the clutch produced between day 12 and day 15 for infected (dark gray) and uninfected (light gray) animals. This clutch represents the first investment in reproduction post-exposure to the parasite but pre-castration. Asterices denote treatments where infected animals had statistically significantly larger clutches than uninfected animals.

**Figure S7:** Survivorship curves for control and exposed animals across the treatments. Colors denote the feeding interval treatment ($T_{1}$: black, $T_{2}$: orange, $T_{3}$: light blue, $T_{4}$: green, $T_{5}$: yellow, $T_{6}$: dark blue).

**Table S1:** Food concentration and feeding interval for each treatment. All treatments received on average 0.6 mgC/vial every six days.

| **Treatment** | **Transfer Food Concentration (mgC/vial)** | **Feeding interval** |
| --- | --- | --- |
| *T_1_* | 0.1 | Every day |
| *T_2_* | 0.2 | Every two days |
| *T_3_* | 0.3 | Every three days |
| *T_4_* | 0.4 | Every four days |
| *T_5_* | 0.5 | Every five days |
| *T_6_* | 0.6 | Every six days |

**Table S2:** Model selection results for growth and reproduction in control animals across feeding treatments. Comparisons are between the every day feeding treatment (T­_1_) and each of the other treatments (T_2-6­)._ The table shows the difference in AIC values (ΔAIC) between a model that allows each treatment to have a separate smooth spline and a model that has a common smooth across treatments. Positive values indicate more support for the common smooth, and thereby no effect of the feeding treatment (relative to the every day feeding treatment).

| **Comparison** | **ΔAIC Growth** | **Treatment effect on growth?** | **ΔAIC Reproduction** | **Treatment effect on reproduction?** |
| --- | --- | --- | --- | --- |
| *T_1_ vs T_2_* | 5.2 | No | -11.7 | Yes |
| *T_1_ vs T_3_* | 22.8 | No | 1.1 | No |
| *T_1_ vs T_4_* | 0.7 | No | -53.0 | Yes |
| *T_1_ vs T_5_* | -27.4 | Yes | -24.9 | Yes |
| *T_1_ vs T_6_* | -27.8 | Yes | -41.5 | Yes |

**Table S3:** Model selection results for the effect of exposure (without infection) on growth and reproduction across feeding treatments. The table shows the difference in AIC values (ΔAIC) between a model that allows each class (uninfected and control) to have a separate smooth spline and a model that has a common smooth across classes. Positive values indicate more support for the common smooth, and thereby no effect of exposure.

| **Comparison** | **ΔAIC Growth** | **Exposure effect on growth?** | **ΔAIC Reproduction** | **Exposure effect on reproduction?** |
| --- | --- | --- | --- | --- |
| Uninfected vs control in *T_1_* | 9.0 | No | -4.1 | Yes |
| Uninfected vs control in *T_2_* | 7.0 | No | -6.8 | Yes |
| Uninfected vs control in *T_3_* | 16.0 | No | 21.1 | No |
| Uninfected vs control in *T_4_* | 16.5 | No | 7.3 | No |
| Uninfected vs control in *T_5_* | 5.3 | No | 2.1 | No |
| Uninfected vs control in *T_6_* | 15.1 | No | 3.2 | No |

**Table S4:** Model selection results for the effect of infection on growth and reproduction across feeding treatments. The table shows the difference in AIC values (ΔAIC) between a model that allows each class (infected and control) to have a separate smooth spline and a model that has a common smooth across classes. Positive values indicate more support for the common smooth, and thereby no effect of infection.

| **Comparison** | **ΔAIC Growth** | **Infection effect on growth?** | **ΔAIC Reproduction** | **Infection effect on reproduction?** |
| --- | --- | --- | --- | --- |
| Infected vs control in *T_1_* | -54.5 | Yes | -186.5 | Yes |
| Infected vs control in *T_2_* | -42.6 | Yes | -133.2 | Yes |
| Infected vs control in *T_3_* | -24.8 | Yes | -38.9 | Yes |
| Infected vs control in *T_4_* | 17.0 | No | -7.1 | Yes |
| Infected vs control in *T_5_* | 0.3 | No | -28.3 | Yes |
| Infected vs control in *T_6_* | 6.9 | No | 11.4 | No |

**Figure S1**


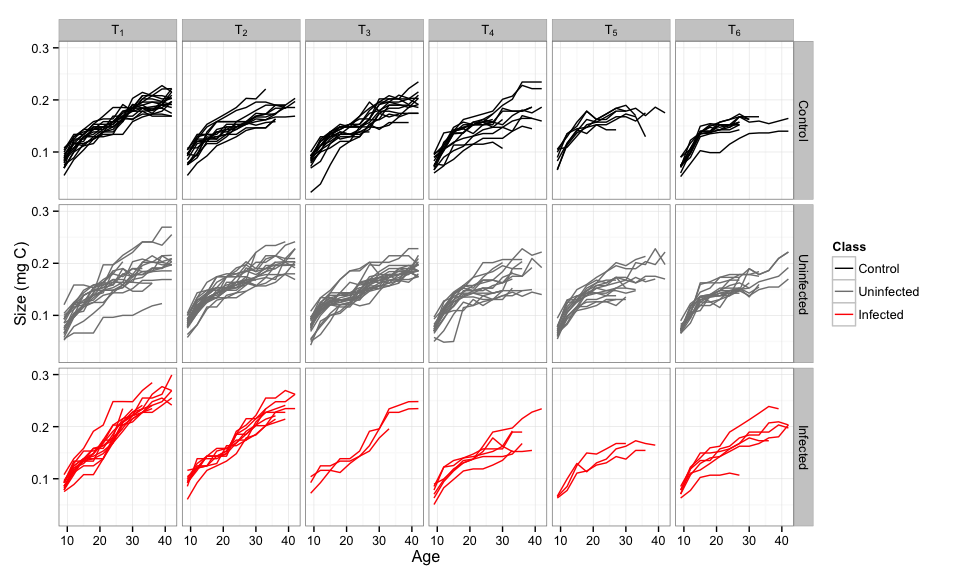


**Figure S2
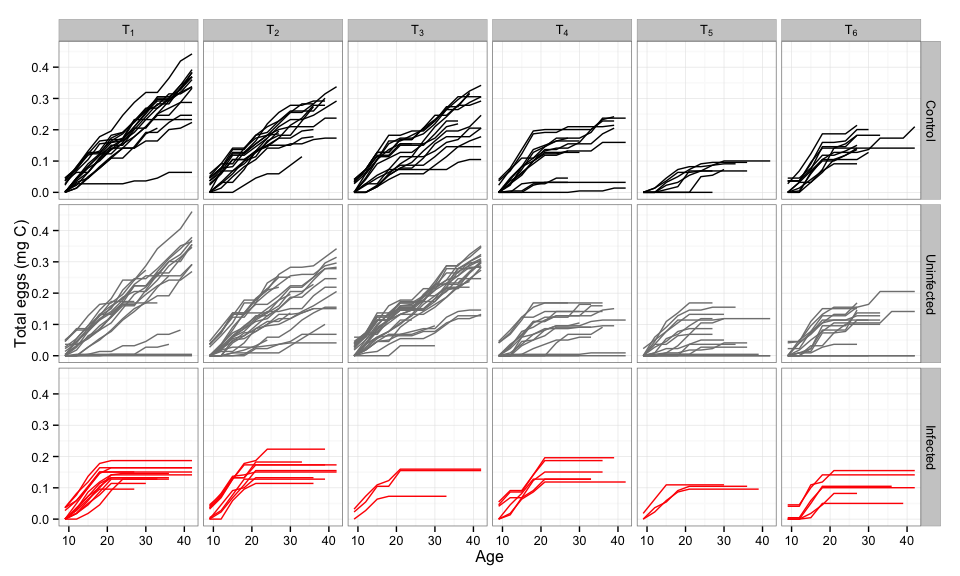
**

**Figure S3**

**
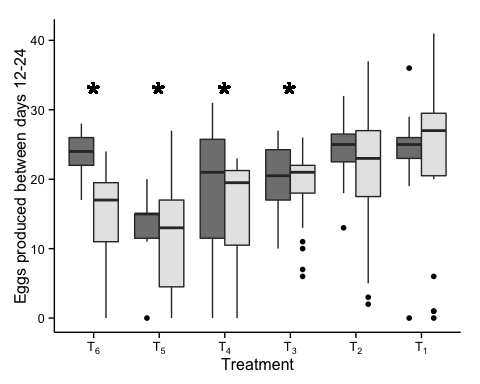
**

**Figure S4**

**
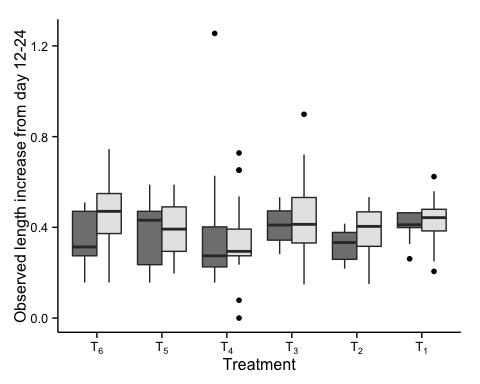
**

**Figure S5**


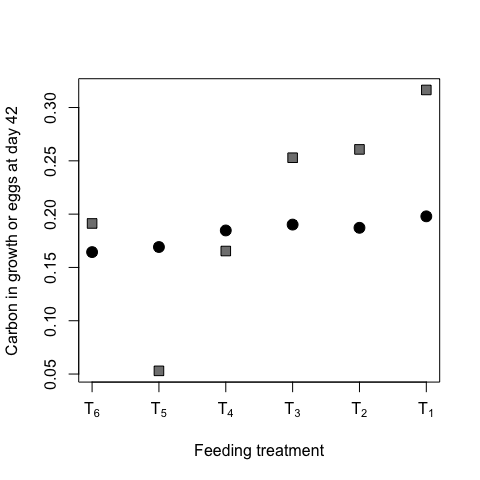


**Figure S6**

**
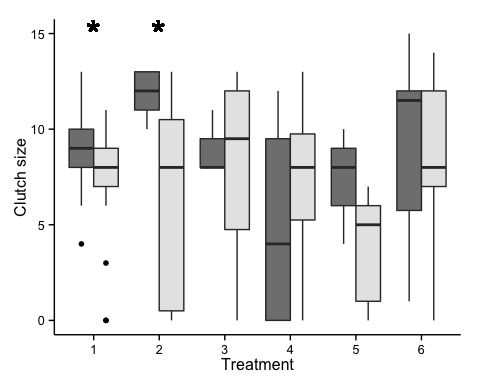
**

**Figure S7**

**
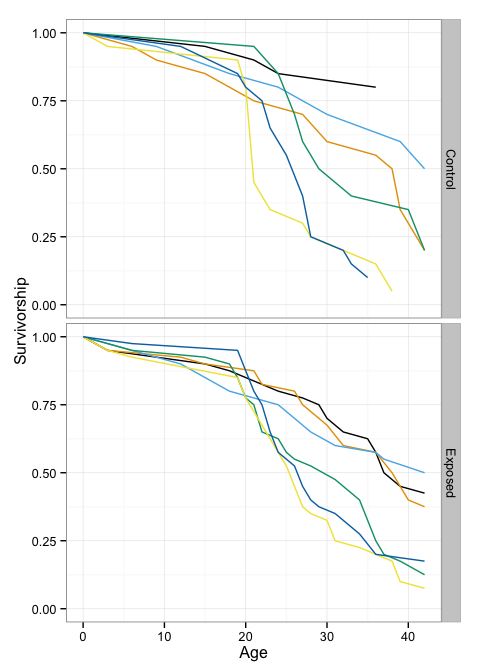
**
